# When to screen for developmental language disorder: a review of age-specific evidence

**DOI:** 10.3389/fped.2025.1646686

**Published:** 2025-10-17

**Authors:** Ji Hyun Park, Min Cheol Chang

**Affiliations:** ^1^ Department of Pediatrics, Brookdale University Hospital and Medical Center, Brooklyn, NY, United States; ^2^ Department of Physical Medicine and Rehabilitation, College of Medicine, Yeungnam University, Daegu, Republic of Korea

**Keywords:** developmental language disorder, age, screening, diagnosis, prediction

## Abstract

Developmental language disorder (DLD) is a heterogeneous condition with challenges in determining the optimal timing for screening. Despite the complexities of early language development, clinical decisions must still be made regarding when to identify children at risk. Recent literature has emphasized the need for the age-specific evaluation of screening precision. This review aims to identify the earliest age for acceptable predictive validity. A narrative synthesis of studies evaluating the validity of DLD screening tools or protocols was conducted, covering ages below 2–4 years. Screening before age 2 demonstrates insufficient sensitivity as a standalone screening point. By age 2.5, several tools achieve sensitivity and specificity above 70%–80%, meeting recommended thresholds. At age 3, screening shows adequate concurrent validity. Screening at age 4 is more aligned with diagnosis than early detection. Based on existing evidence, 2.5 years is the earliest age at which DLD screening tools begin to demonstrate acceptable predictive performance. The findings may inform clinical guidelines on DLD screening and highlight the need for further age-stratified studies to refine DLD screening strategies.

## 1 Introduction

Developmental language disorder (DLD) is a condition in which children experience persistent difficulties in using or understanding spoken language without a known biomedical cause. Following the Criteria and Terminology Applied to Language Impairments: Synthesizing the Evidence (CATALISE) consensus project, DLD has replaced specific language impairment (SLI) as the preferred term to ensure consistency in terminology across clinical and research fields (1). In line with this recommendation, this review uses the term DLD even when referring to studies that originally used the term SLI. The CATALISE project also removed rigid exclusion criteria; specifically, the presence of neurobiological or environmental risk factors no longer precludes a DLD diagnosis, and DLD can co-occur with other neurodevelopmental disorders. The diagnosis also does not require a mismatch between verbal and nonverbal ability.

DLD is a prevalent neurodevelopmental condition among preschool children. A systematic review commissioned by the United Kingdom National Health Service synthesized 16 prevalence estimates and reported rates of primary speech and language delay ranging from 2% to 19% among children younger than 5 years (2). A subsequent population-based survey in England reported a DLD prevalence of 7.6% among children aged 4–5 years (3). This wide range of prevalence reflects a key challenge in DLD identification, i.e., the lack of consensus on diagnostic cutoffs (4). Different cutoff criteria are used to define language impairments, as there is no consensus on the distinction between impaired, delayed, and typical language development (1, 4). Most commonly, a vocabulary size below the 10th percentile for age has been used to identify late-talking toddlers (5). Expressive vocabulary of fewer than 50 words or the absence of word combinations at age 2 is another commonly used criterion for defining late-talking toddlers (6).

Early identification of children at risk for DLD is complicated by the high variability in early language development. Longitudinal studies, such as the Australian Early Language in Victoria Study, have shown that many late talkers catch up developmentally by school entry; however, some children who initially demonstrate typical language development go on to exhibit persistent impairments (7). This variability has prompted caution regarding universal, one-time screening. Nevertheless, it highlights the need for screening measures with both high sensitivity and specificity to effectively distinguish transient delays from persistent language disorders. The American Academy of Pediatrics (AAP) recommends ongoing developmental surveillance supplemented by standardized screening tools (8), which can improve early identification and facilitate timely intervention.

An increasing body of evidence underscores the multifactorial etiology of DLD, which involves both genetic and environmental factors. Twin and family studies consistently indicate a heritable component, with higher concordance among monozygotic twins compared with dizygotic twins (9). Several candidate genes, including *FOXP2*, *CNTNAP2*, and *ATP2C2*, have been implicated in language development and may contribute to DLD risk (10). However, genetic predisposition alone does not fully explain the disorder. Perinatal complications, low birth weight, and otitis media with effusion may affect language acquisition (11). In addition, psychosocial factors, such as parental education, language exposure at home, and the overall richness of the linguistic environment, can influence the rate and trajectory of language development (12). Not surprisingly, screen exposure shows a dose-response relationship with developmental delays in communication (13), likely due to reduced language-rich interactions. Therefore, DLD is best conceptualized as a multifactorial condition arising from complex gene-environment interactions.

The neurodevelopment of language-related brain regions closely parallels early language acquisition. Broca's area and Wernicke's area, linked via the arcuate fasciculus, undergo rapid maturation between ages 2 and 5 years, coinciding with the most pronounced gains in vocabulary and syntax (14). Neuroimaging studies of children with DLD have reported the reduced integrity of the arcuate fasciculus and hypoactivation in the left frontal and temporal regions (15, 16), suggesting that atypical neural maturation underpins the disorder and supporting the rationale for screening within this critical developmental window.

Age is critical in understanding language development and delay (17). Although early identification of DLD is widely supported, there is no clear consensus on the optimal age for screening. Many clinicians have agreed that the preschool period is important for screening DLD. However, the use of a broad age range (younger than 5 years) for synthesizing studies may mask important differences associated with age at screening (17). Determining the optimal age for screening has been a complicated topic due to extreme variability in language development trajectories among young children. The main challenge with screening too early is the high rate of spontaneous improvement in initially delayed children, making it difficult to predict delay persistence. Conversely, delayed screening risks missing the optimal intervention window for children with persistent language difficulties. Furthermore, late-onset cases with no initial delay further complicate the screening timeline (18). Despite these complexities, clinical decisions regarding the appropriate timing of screening and diagnosis of DLD are required to ensure that children who need intervention are identified promptly for the best language outcome (4).

In 2021, Sansavini et al. conducted a comprehensive scoping review that examined the evidence surrounding the optimal screening age for DLD. Although the review did not identify a specific optimal age, it suggested that the period between 2 and 3 years may be the most appropriate (19).

This narrative review seeks to synthesize age-specific evidence regarding the optimal age for screening DLD across key ages.

## 2 Performance standards for developmental screening

The AAP recommends sensitivity and specificity levels of at least 70%–80% as acceptable for developmental screening tests (20). Due to challenges inherent in measuring child development, these values are lower than generally accepted for medical screening tests (20). Trade-offs between sensitivity and specificity are inevitable when establishing cutoffs for standardized tests. Specificity may be preferred to minimize false positive results, thus avoiding unnecessary parental concern and overuse of intervention services (21). Conversely, compromising sensitivity risks missing the critical intervention window. Therefore, it is essential to conduct DLD screening at an age that can reliably achieve satisfactory performance in both sensitivity and specificity.

Although some researchers have argued that reduced specificity may be acceptable in language screening if it allows earlier identification of children at risk for persistent DLD, our review underscores the importance of achieving both high sensitivity and specificity to ensure accurate detection and minimize unnecessary referrals (22).

## 3 Screening before age 2

Screening before age 2 presents significant limitations in predictive accuracy. In a large-scale cohort study (*N* = 3,759), Henrichs et al. (23). examined the predictive validity of expressive vocabulary scores from the MacArthur Short Form Vocabulary Checklist (CDI-N) at 18 months for predicting Language Development Survey (LDS) delay status at 30 months, with delay defined at the <10th percentile cutoff (Table 1). The receiver operating characteristic (ROC) curve showed a moderate predictive performance (AUC = 0.74). The correlation between CDI-N word production at 18 months and LDS word production at 30 months was low (0.34), indicating only a modest degree of association between the two measures. Furthermore, sensitivity (30%) and positive predictive value (PPV: 29%) were very low. A low sensitivity indicates a high false negative; most children who were delayed (below the 10th percentile) at 30 months initially scored in the normal range (above the 10th percentile) at 18 months. This finding suggests that expressive vocabulary at 18 months has insufficient predictive power for detecting actual cases of later language difficulties.

**Table 1:** Age-specific evidence on DLD screening accuracy.

Age at screening	Study	Tool	Cutoff	Sensitivity	Specificity	Note
18 months	Henrichs et al. (23)	CDI-N	<10th percentile	30%	93%	Low sensitivity
24 months	Feldman et al. (28)	CDI-WS	<10th percentile	50%	90%	Limited sensitivity
	Sim et al. (29)	ELFRA-2	Tool defined <50 words or 50–80 words + low grammar	61%	94%	
30 months	Nayeb et al. (30)	Swedish national protocol	Protocol-based	Monolingual: 91% Bilingual: 88%	Monolingual: 91% Bilingual: 82%	Adequate sensitivity and specificity
	Yasumitsu-Lovell et al. (31)	ESSENCE-Q	Score of ≥3/11	84.9%	84.8%	
36 months	Holzinger et al. (33)	SPES-3	ROC-based cutoff	87.8%	87.6%	Adequate sensitivity and specificity
	Westerlund et al. (34)	Municipal protocol	Protocol-based	77.3%	99%	

CDI-N, MacArthur short form vocabulary checklist; CDI-WS, MacArthur-Bates communicative development inventory-words and sentences; ELFRA-2, German adaptation of MacArthur-Bates communicative development inventory toddler form; ESSENCE-Q, early symptomatic syndromes eliciting neurodevelopmental clinical examinations questionnaire; SPES-3, Sprachentwicklungsscreening; ROC, receiver operating characteristic.

The low sensitivity and PPV of screening before age 2 are consistent with findings from other studies (24, 25). In contrast, specificity appears to be high at this age, which was reported as 93% by Henrichs et al. (23), 93% by Thal et al. (25), and 90% by Westerlund et al. (26), suggesting that early screening is more effective at identifying children at risk. However, as the observed sensitivity falls below the AAP's recommended threshold of 70%–80% (19), screening before age 2 does not meet the criteria for a reliable single screening point.

Neurodevelopmentally, the limitations align with the maturation timeline of language-related brain regions. After age 2, Broca's area, Wernicke's area, and the arcuate fasciculus undergo rapid maturation, characterized by increased lateralization and strengthened connectivity via the arcuate fasciculus (14, 27). This developmental progression allows many children with early delays to “catch up”, complicating efforts to distinguish transient delays from persistent disorders for children under age 2.

## 4 Screening at age 2

Screening at age 2 also shows limited predictive power, with no apparent improvement in sensitivity compared to that prior to age 2. Feldman et al. (28). reported modest predictive metrics using the MacArthur-Bates Communicative Development Inventory-Words and Sentences (CDI-WS) at age 2 by applying a cutoff at the 10th percentile on three expressive language subscales (Vocabulary Production, Three Longest Sentences, and Sentence Complexity) (Table 1). When validated against five direct lab-based assessments at age 3 (McCarthy GCI, McCarthy Verbal scale, PPVT-R, and number of different words and mean length of utterance from parent-child conversations), the CDI-WS achieved a sensitivity of 50% and specificity of 90%.

Furthermore, a systematic review by Sim et al. (29). demonstrated the limitation of screening at age 2. The review examined a total of 11 studies that reported the predictive validity of preschool developmental screening; 6 studies focused on language-only screening tools. One of the strongest tools identified was the German adaptation of the MacArthur-Bates Communicative Development Inventory Toddler Form (ELFRA-2) administered at 24 months and followed up at 37 months using the SETK 3-5, a German standardized language test. This tool achieved a sensitivity of 61% and specificity of 94%.

By age 2, children typically demonstrate substantial gains in language development, with increased vocabulary size, emergence of two-word combinations, and early grammatical structures. Despite this maturation, screening at this age remains limited in predictive capacity. Some children are still in a transitional phase during which late talkers may rapidly improve without intervention. Consequently, screening at age 2 offers high specificity but unsatisfactory sensitivity across tools, limiting its reliability as a standalone screening point for DLD.

## 5 Screening at age 2.5

Screening performance at age 2.5 begins to show satisfactory sensitivity and specificity. In Sweden, the typical language screening age has been lowered from 3 to 2.5 years since 2016. A study by Nayeb et al. (30). supported this policy shift by demonstrating diagnosis stability from 2.5 to 3 years of age (Table 1). The study included 141 children screened according to Sweden's national health protocol; 93 of them were bilingual, reflecting the country's diverse demographics. Initial screenings were conducted by nurses, with diagnostic confirmation by a speech-language pathologist. For monolingual children, the screening protocol achieved a sensitivity of 91% and specificity of 91%. For bilingual children, the sensitivity and specificity were 88% and 82%, respectively. The diagnostic performance of the study protocol met the AAP's screening thresholds for both monolingual and bilingual children. These findings support the feasibility of language screening at 2.5 years.

Although not specific to language delay, additional evidence from the study by Yasumitsu-Lovell et al. (31). using the ESSENCE-Q neurodevelopmental screening tool highlights the value of early screening at age 2.5. The findings suggest that the use of ESSENCE-Q at age 2.5 may serve as an effective screening tool for neurodevelopmental disorders, including language delay, by age 3. The 11-item tool, originally developed by Gillberg (32), is based on the ESSENCE concept (Early Symptomatic Syndromes Eliciting Neurodevelopmental Clinical Examinations) and covers multiple developmental domains including language, motor, sensory, and social functions. When validated against a comprehensive clinical diagnosis at age 3, ESSENCE-Q demonstrated strong predictive validity. Using a score cutoff of ≥3 in ESSENCE-Q, ROC curve analysis yielded an AUC of 0.91, with 84.9% sensitivity and 84.8% specificity. Although ESSENCE-Q is not a language-specific screening tool, its strong predictive validity reinforces the utility of multi-domain tools in identifying children at risk for DLD and other neurodevelopmental disorders.

Overall, the evidence supports age 2.5 years as the point at which screening performance consistently demonstrates satisfactory levels of sensitivity and specificity. In particular, the Swedish policy shift and supporting data from both monolingual and bilingual populations underscore the effectiveness of screening at 2.5 years (30). Moreover, the use of multi-domain tools such as ESSENCE-Q at this age can further strengthen early identification by capturing comorbidities that commonly co-occur with DLD.

## 6 Screening at age 3

Although predictive validity is a key criterion in determining the efficacy of a screening tool (29), there appears to be a lack of predictive screening studies specifically at age 3. However, a few studies have reported concurrent validity at this age, supporting the feasibility of using certain tools to identify DLD at age 3 (Table 1) (33, 34).

Holzinger et al. (33). evaluated the SPES-3 (Sprachentwicklungsscreening) tool for identifying DLD among 3-year-old children within primary care in Austria, selecting age 3 based on evidence showing that grammatical competence is a good marker of language development at this stage. The tool combines parent-reported subscales (expressive vocabulary and expressive grammar) and pediatrician-administered subscales (noun plural production and sentence comprehension). The parent-reported subscales showed the highest diagnostic accuracy (AUCs of 0.908 for expressive vocabulary and 0.910 for expressive grammar) compared with the accuracy of the pediatrician-administered subscales (AUCs of 0.816 for noun plural production and 0.705 for sentence comprehension). Integrating parent-reported subscales into a composite screening score further increased diagnostic accuracy, achieving an AUC of 0.946. At the cutoff yielding the most balanced test performance, the sensitivity and specificity were 87.8% and 87.6%, respectively.

Westerlund et al. (34) assessed a municipal screening protocol administered at age 3 within the Swedish health system. The protocol incorporated structured nurse interviews, parent questionnaires, and direct assessments of expressive and receptive language skills. In a subset of children who received same-age diagnostic confirmation by a speech-language pathologist, the sensitivity and specificity were 77.3% and 99%, respectively.

Overall, these findings suggest that both SPES-3 and the municipal screening protocol may be adequate diagnostic screening tools, appropriate for use at age 3.

## 7 Limited utility of screening at age 4

To the best of our knowledge, there is no predictive screening study targeting ages 3–4. By age 4, it is known that language development has typically reached a level of stability that allows for the diagnosis of DLD. A recent scoping review concluded that by age 4, a diagnosis of DLD could be made (19). Klem et al. (4). reported strong longitudinal stability in the language skills of children from age 4 to 6, with high-performing and low-performing children showing parallel growth trajectories that preserved initial differences. Given that the primary goal of screening is to identify children at risk before difficulties consolidate, the utility of screening at age 4 is limited.

## 8 Summary and discussion

Our narrative review identifies age 2.5 years as the earliest age at which DLD screening tools consistently achieve acceptable predictive validity, meeting the AAP's recommended thresholds for sensitivity and specificity (≥ 70%–80%). Notably, the Swedish national screening protocol at this age achieved 91% sensitivity with 91% specificity for monolingual children and 88% sensitivity with 82% specificity for bilingual children, demonstrating both diagnostic accuracy and feasibility in a public health setting.

Beyond on-time screening, developmental surveillance, defined as the ongoing monitoring of developmental milestones, parental concerns, and risk factors during routine well-child visits, is a vital complementary strategy. Research indicates that combining surveillance with standardized screening outperforms either approach alone, improving detection rates and facilitating early referral to intervention services (35).

Furthermore, parent-report screening tools have emerged as practical and effective alternatives. For example, the SPES-2 model employs a two-stage approach: an initial parent-reported questionnaire (covering expressive vocabulary, two-word combinations, and parental concerns), followed by direct pediatric assessment only for children who fail the initial screen. In a previous study, this model demonstrated high diagnostic accuracy (AUC = 0.885, sensitivity = 0.74, specificity = 0.86) and was rated as highly feasible by pediatric providers (36).

Supporting a stepped care approach, a combination of parent- and provider-administered tools for children around age 3 has shown high diagnostic accuracy while remaining concise enough for integration into routine preventive care, even when implemented by non-specialist clinicians (33).

Although language screening at 2.5 years provides a strong foundation for early detection, integrating multiple approaches—developmental surveillance, parent-report instruments, and tiered diagnostic pathways (e.g., initial proxy screening followed by direct assessment)—offers a more flexible and efficient framework. This multi-tiered strategy can enhance early identification, streamline clinical workflows, and ensure timely evaluation and intervention for high-risk children.

## 9 Limitations

A key limitation of this review is the small number of available studies on the predictive validity of screening across different ages (19). Additional age-stratified studies are warranted to refine clinical recommendations regarding the optimal timing for DLD screening.

## 10 Conclusion

At and below age 2, sensitivity remains insufficient to identify children with true language impairment. At age 2.5, screening tools begin to demonstrate predictive accuracy, with both sensitivity and specificity reaching 70%–80%. The use of multi-domain instruments such as ESSENCE-Q appears to be particularly effective for screening at this age. At age 3, although some tools show adequate concurrent validity, data on predictive validity remain limited. As persistent DLD stabilizes by age 4, diagnosis can often be confirmed at this age. Current evidence suggests that 2.5 years is the earliest age at which DLD screening tools demonstrate adequate predictive power.
